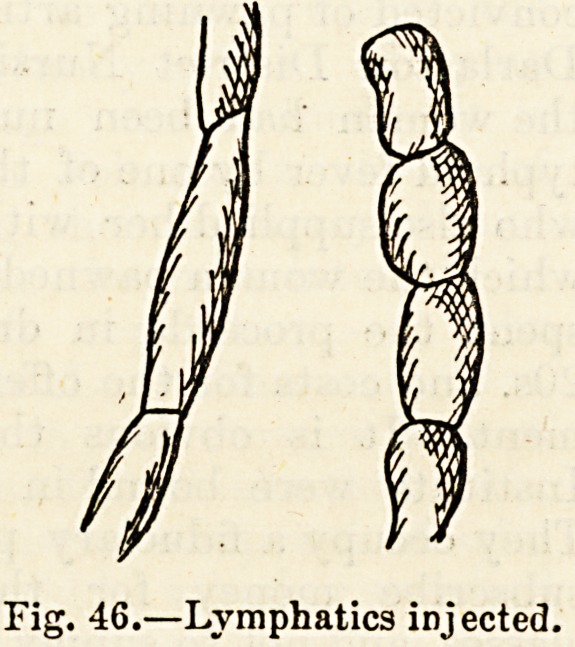# The Hospital. Nursing Section

**Published:** 1902-06-14

**Authors:** 


### The Hospital.

### Nursing Section.

Contributions for this Section of "The Hospital" should be addressed to the Editor, "The Hospital" Nursing Section, 28 & 29 Southampton Street, Strand, London, W.C.

No. 820.—Vol. XXXII. *SATURDAY, JUNE* 14, 1902.

### Notes on News from the Nursing World.

### THE NURSES' HOME AT GUY'S HOSPITAL.

The Prince and Princess of Wales have graciously intimated to the Governors of Guy's Hospital their intention of opening the Henriette Raphael Nurses' Home on Monday, July 7th, at 3.30 p.m.

### THE KING AND THE NURSES' HOME.

A remarkable illustration of the personal interest which the King takes in all that appertains to the welfare of nurses is afforded by a letter written by the direction of his Majesty to a Barry paper. The letter states that "all movements which have for their object the alleviation of human suffering have his Majesty's hearty approval," and expresses a hope "that the efforts on the part of the Barry Council to raise funds by voluntary subscriptions to defray the debt on the Queen Victoria Jubilee Nurses' Home by way of celebration of the Royal Coronation will be successful." The people of Barry have been happily put on their mettle by the King, and we do not doubt that they will rise to the occasion.

### THE ROYAL RED CROSS FOR NURSES AT PRETORIA.

There was an interesting ceremony at Pretoria on Sunday morning prior to the great Thanksgiving Service in the Church Square. About ten o'clock Lord Kitchener and his Staff from the Government Buildings appeared on the platform, and having presented several officers and men with the Victoria Cross, the Commander-in-Chief proceeded, amid many signs of enthusiasm, to bestow the Order of the Royal Red Cross upon eleven nursing sisters. The scarlet facings of the nurses was in striking contrast with the prevailing khaki hue. As soon as the ceremony was over the Thanksgiving Service commenced.

### AN INCOMPLETE VOTE OF THANKS.

The two Houses of Parliament have very properly passed a vote of thanks to the Imperial forces for the manner in which they discharged their duties in South Africa; but we are not surprised that nurses are commenting on the fact that no reference was made to the work of the army sisters, either in the long resolution embodying the vote, or in the sufficiently exhaustive speeches delivered. It is true that the services rendered by the doctors and surgeons were also ignored in the same way, but this is no consolation to the friends of the numerous nurses whose labours were so highly valued by the troops. We cannot understand why it was not thought necessary and desirable to include in the vote of thanks by the Legislature those who, though non-combatants, are just as much entitled as the men who fought on the field to some expression of national appreciation.

### ROYAL NATIONAL PENSION FUND FOR NURSES.

It is appropriate in our Coronation number to refer to the growth of the great movement for the benefit of nurses, of which the King is patron and the Queen president. We allude, of course, to the Royal National Pension Fund, which owes so much of its amazing success to the support it has received from their Majesties. The progress of the fund between 1890 and 1895 was very striking, but far more remarkable were the strides made between 1895 and 1901. Thus, while the number of policies issued to December 31, 1895 was 4,588, it was 9,141 to December 31, 1901. The premiums received in 1895 amounted to £45,571, while in 1901 they reached the sum of £81,000; the total income, which was £54,355 in 1895, advanced to £105,000 in 1901, and the total invested funds leaped from £256,556 to £641,000. Moreover, the fact that last year the contributions from, or for, nurses, came to nearly five times as much as in 1890, the total income for the year being more than five times greater, and the invested funds over eight and a half times more, is emphasised by the circumstance that upwards of £120,000 has been returned to nurses who had to give up membership, in addition to over £11,000 distributed in sick pay. Comparing the figures for the years between 1890 and 1895 and 1895 and 1901, we find that the increase of the policies issued in the later period was equal to 752 a year, the increase in the premiums received £5,905 a year, the increase in the total income £8,441 a year, and the increase in the funds £64,000 a year. This is the average, but in 1901 the increase in the premiums received actually amounted to over £6,000, and the increase in the invested funds was nearly £72,000. In other words, last year showed all round a more extraordinary development than any preceding twelve months, and yet there is every reason to believe that the high-water mark of the fund has still to be reached.

### THE PENSION FUND ARMLET.

As several of our readers who are Queen's nurses, and also belong to the Royal National Pension Fund for Nurses, have asked us how the Pension Fund armlet should be worn with the Queen's brassard, we have pleasure in informing them that it should be placed on the left arm below the brassard of the Queen's Institute.

### THE NEW PHASE OF THE ATTACK ON NURSES.

The title of the reply of Miss M. F. Johnston to the criticism provoked by her original attack upon nurses is a tacit confession of her failure to make good her case. Her first article was supposed to be directed against hospital nurses. Now, having realised how entirely her animadversions in that direction have been disproved, she attempts to deal with "the question of the modern trained nurses." This is a much larger issue, and it is obvious that nurses who are not amenable to the discipline and influence of hospital life may, either from lack of character, or from want of care, reflect discredit upon their calling, instead of adorning it. But no lucubrations from Miss Johnston were needed to prove that among the enormous number of modern trained nurses attached to homes, or working on their own account, there is a small percentage of black sheep. As for the cases which Miss Johnston mentions in support of her sweeping charges, we observe that one only is that of a patient in a London hospital, who had to ask a ward sister to make her pillows comfortable because two nurses declined to alter them on the ground that any alteration would spoil the appearance. This is indeed a formidable basis for the indictment of hospital nurses as callous and indifferent! The remainder of Miss Johnston's personal friends who are alleged to have suffered from the offences of nurses were private patients. These instances would have merited more attention if Miss Johnston had been courageous enough to give chapter and verse for her statements. We know so well the stories about smart nurses from homes pestering male patients with their attentions, but we should really like to learn where the nurse who said that "the funny noises" of dying people nearly made her laugh was trained. It is refreshing to turn from Miss Johnston's citation of cases—which, even assuming their accuracy, do not justify her effort to promote the best interests of nurses by blackening their reputation—to the interesting articles by Miss Lucy Rae and Mrs. Alec Warde, also appearing in the current issue of the *Nineteenth Century*. Miss Rae thinks that if the nursing profession were entirely composed of educated gentlewomen, there would be a higher standard of character—a very debatable point; while Mrs. Warde avers that the private nurse takes often too much upon herself in the sick-room. This may be true, but here again the fault as frequently lies with the friends of the patient as with the nurse. The controversy necessarily caused by Miss Johnston's attack will not injure those against whom it was levelled.

### ENGLISH NURSES AND BOER REFUGEES.

We recently published a couple of articles, by Miss Brereton, describing the duties of the nurses in the concentration camp at the time the Ladies' Commission visited South Africa. This week an English sister, writing with the permission of the authorities, sends us an account of nursing in the camps at Krugersdorp up to date. It will be seen that the work of the nursing staff is fortunately no longer onerous, and that ample provision is made for their recreation. The concentration camps will now, of course, soon be things of the past, but we can quite understand that the refugees are, as a rule, perfectly willing to remain where they are until a proper system of supply depôts has been established for them in the outlying districts. It is satisfactory to think that they will carry away pleasant recollections of the sisters.

### MADRAS GENERAL HOSPITAL.

A number of grievances under which it was alleged the nurses of the Madras General Hospital suffer having been recited in the columns of a Madras journal, the authorities have denied the accuracy of several statements. They admit that the meat supplied to the nursing staff is occasionally tough; that enamelled ware is partially used for the purpose of reducing the nurses' breakage bills; and that the nurses are required to defray a proportion of the cost of linen lost. If there are really no other foundations than these for dissatisfaction, it should not be difficult to allay discontent.

### ODDFELLOWS AND THE NURSING MOVEMENT.

The unsolicited testimony of the great organisations of Oddfellows, with regard to the work of nurses is always valuable. At the monthly meeting of the Darwen Lodge of Oddfellows, Brother Duxbury read a paper on the local nursing organisation, in the course of which he said: "I assert that this Association is doing God's greatest work. The lives of these nurses, unless they are really and truly interested in the noble work they have set themselves cannot be happy lives, and I can conceive of no personal motives to induce them to engage in such work, except that it is congenial to their natures, and in proportion as this element enters into their work as with all other forms of work, so will success and satisfaction to their patients and to themselves be." This panegyric which Mr. Duxbury confessed was the result of some little investigation into the history and methods of the Association, was endorsed by the company present, and his appeal to the people of Darwen to interest themselves in the organisation, and to take a share in its management, was warmly applauded.

### ROYAL BRITISH NURSES' ASSOCIATION.

Her Royal Highness Princess Christian will preside at the annual meeting of the Royal British Nurses' Association, to be held at the Imperial Institute on Monday next, June 16th, at 12 noon. A meeting of the General Council of the Royal British Nurses' Association will be held at 10 Orchard Street, on Friday, June 13th, at 5 p.m., when we hope that a conciliatory spirit will prevail. Many of the best friends of the nursing profession, who have recognised how very one-sided the training of the midwife must be as compared with that of the nurse, have expressed some surprise that the Royal British Nurses' Association should have obtained, or wished for, representation on the proposed midwives' board; and that position having been offered, it would, we think, come with somewhat of an ill grace from them to attempt, even before they have taken their seat, to interfere with other clauses of the Bill to which they owe their new dignity.

### THE VALUE OF OBSERVATION.

Striking testimony is given by Dr. James Cantlie in this month's *Charing Cross Hospital Gazette* to the value of observation in clinical medicine and surgery. He takes as his text a certain Nurse M—— from whom he admits that when clinical clerk to one of the doctors 30 years ago he learnt much, because she was careful to notice every change in the patient, however small, and never considered anything too trivial to be recorded. When first carbolic acid was being tried for malignant disease of the stomach, and the doctors were not certain whether a rash which ensued was the direct result of the treatment, she was able to inform them of three separate patients who had been under her care in the hospital who, when being treated with carbolic, had all developed a similar rash, and also to show them by her note-book that the temperature of patients who died from acute rheumatism usually rose after death. Dr. Cantlie recommends the present students of Charing Cross Hospital to keep their ears open and try and find a Nurse M—— for themselves, so that "they may benefit by the experience to be gained in the wards of a large hospital by an observant nurse."

### NURSES' DAY IN SOMERSETSHIRE.

A very pleasant gathering of nurses and others interested in their work has recently taken place in Somersetshire. A similar meeting was held last year at the Grange, Kingston, and was so thoroughly appreciated as to encourage its promoters—among whom Miss E. L. C. Eden was the chief—to repeat it, and perhaps to make it permanent. It was felt that the position of a nurse working in a country district, or in a small cottage hospital or union infirmary, is often a very lonely one, and that she has few opportunities of keeping herself in touch with the nursing world, or with the new discoveries and improvements always being made; and it was thought that if all the nurses in the county could meet together occasionally for social purposes and to listen to an address on some of the difficulties of a nurse's life, it would both tend to diminish this feeling of isolation and also be a real help to them in their work. This year the meeting, by the kind invitation of Mrs. Martin Gibbs, was held at Barrow Court, near Flax Bourton, on May 29th. There were about 100 nurses from all parts of the county present. The beauty of the grounds, with their terraced walks, and avenues of yew, and the wide lawns planted with flowering shrubs, backed by the old stone house with its mullioned windows, would have repaid a longer journey than that undertaken even by those who had come from the furthest corners of the county. But there were also the other attractions of tea out-of-doors, and music and acting in the fine old tithe barn. A collection of nursing requisites, kindly lent by some of the leading firms, likewise proved a source of considerable interest. An address given by Miss Amy Hughes provoked much enthusiasm, and a hearty vote of thanks was accorded to her, as well as to Mrs. Gibbs and the other ladies who had helped to make the meeting a success. A suggestion by Miss Hughes, that small groups of nurses should meet together in different parts of Somersetshire, thus increasing and continuing the enjoyment of the larger gatherings, was warmly applauded.

### "THE MOST DIFFICULT OF ALL PROBLEMS."

The Peckham Nursing Association has derived considerable benefit from a drawing-room sale of work which has just been held on its behalf. It is estimated that after all expenses have been paid over £40 will be realised. The association only completed the first year of its existence last October, but it has already rooted itself in the affections of the residents, and on both of the sale days several speakers described it as a great boon. Mr. Hinchcliffe went so far as to observe that it had solved "the most difficult of all problems—where to find a really good nurse outside a hospital." The work of the nurse is so highly appreciated that it is in contemplation to start a second as soon as the funds will permit.

### NURSES' UNION.

The annual sale of work for medical missions and social afternoon was held by Miss Dashwood on Friday last week at the Central Institute, 26 George Street, Hanover Square. As each nurse entered she was presented with a button-hole of lilies of the valley to wear. Lady Spencer Churchill and her daughter came for a short time. Lady Spencer sang three songs: "Oh, dry those tears," "The sweetest flower that blows," and "Violets." The accompaniments were played by Miss Churchill, and between the songs the latter gave three recitations. Miss Edith Dashwood sang several songs. Miss Constance Dashwood gave two recitations, and Miss Wills, late Zenana Mission nurse, who wore native costume, a short account of her work abroad. Three nurses were down in the programme to sing, but one had to go early, the second had a cold, and the third sang "The Gates of the West." £10 15s. was realised by the sale of work, which will pay for the bed supported by the Nurses' Union.

### PAWNING THE PROPERTY OF A NURSING INSTITUTE.

We are glad to see that the Wednesbury magistrates have adequately punished Ellen Poole, who was convicted of pawning articles supplied to her by the Darlaston District Nursing Institute. A child of the woman had been nursed during an attack of typhoid fever by one of the nurses of the institute, who also supplied her with sheets and bed clothing, which the woman pawned at a shop in the town and spent the proceeds in drink. She has been fined 20s. and costs for the offence, or a month's imprisonment. It is obvious that the committee of the Institute were bound in such a case to prosecute. They occupy a fiduciary position to the public who subscribe money for the benefit of the working classes, and not to supply means for people to indulge in drink. Happily, incidents of this kind are very rare, for the frequent abuse of a beneficent charity would tend to close the pockets of the benevolent.

### SHORT ITEMS.

The foundation stone of the new Nursing Home in the city of Ripon, which is being erected by public subscriptions in commemoration of the golden wedding of the Marquis and Marchioness of Ripon, will be laid by the Mayoress on Coronation Day. This will form not the least interesting event in the city's celebrations.—The following arrived from South Africa on board the *Dilwara* on the 5th inst.:—Sisters L. A. Bailey, A. M. Read, M. Cameron, and M. Goldsmith. It is stated that all these will return to South Africa. Sisters D. A. Snell and E. C. Humphreys were invalided home on three months' leave.—The south block of the new premises of the Nurses' Hostel Company are now completed, and will be on view to invited guests next Monday between three and seven.—Miss M. Rowland, who died at Neath on the 25th of May, has left the sum of £2,000 to build a home for the Queen's nurses in the town, or to be invested and the proceeds applied to the general purposes of the Neath Nursing Association.—On Saturday, June 21st, the staff of the West Ham Hospital, Stratford, will have an "At Home," and a collection will be made in aid of the funds for the Coronation festivities.

### Lectures to Nurses on Anatomy.

By W. Johnson Smith, F.R.C.S., Principal Medical Officer, Seamen's Hospital, Greenwich.

LECTURE XIX.—THE LYMPHATIC SYSTEM.

In most parts of the living body the different tissues are kept moist and soft and are in part nourished by a thin watery fluid called *lymph*, derived from the fluid part—the plasma—of the blood, and transuded through the thin walls of the capillary blood-vessels. As the drainage from the minute blood-vessels persists, the transuded lymph is removed or *absorbed* from the tissues and is again mixed with the blood.

During the digestion of food a large quantity of milk-like fluid, known as *chyle*, is absorbed from the small intestine, and together with the clear lymph is poured into large veins at the root of the neck to renew and nourish the circulating blood.

These two different processes, the absorption of pure lymph and the absorption of thick milky chyle, are effected by similar thin-walled vessels resembling small veins. Those which take up or absorb lymph are called *lymphatics or absorbents*, those which are connected with the intestine, from the milky appearance of the fluid they contain during digestion, are called *lacteals*.

Both lymphatics and lacteals are associated with a large number of soft rounded bodies, some almost invisible to the naked eye, and most of them varying from the size of a

Fig. 45.—Valves of lymphatics.

Fig. 46.—Lymphatics injected.

small seed to that of an almond—the *glands* of the lymphatic system. Those in connection with the lymphatics are called *lymphatic glands*, and those with the lacteals, from their situation in the mesentry, the membrane suspending the intestinal canal to the spine, are called *mesenteric* glands. The lymphatic glands are few and scattered in the limbs, more abundant in the neck, arm and groin, and are accumulated in large irregular groups in the abdominal and thoracic cavities.

The transparent lymph which, like blood, "sets" or coagulates when shed outside the body, flows through the lymphatics in the same direction as the venous portion of the circulation blood, passes through the lymphatic glands corresponding to the region of the body from which it is absorbed, is poured into a long tube of the size of a goose quill, known as the *thoracic duct*, by which it is conducted along the front of the spine through the abdominal and thoracic cavities, and finally discharged into the circulating stream of blood at the junction of two large veins—the jugular from the head, the subclavian from the upper extremity—on the left side of the neck. The current in the thoracic duct is supplied by lymph from all parts of the body that are drained by lymphatics, except the chest, arm, and head and neck on the right side which discharge their lymph through a short conduit called the *right lymphatic duct* into the junction of the jugular and subclavian veins on the corresponding side.

The fluid absorbed by the lacteals, which during digestion is opaque and milky, and at other times is clear lymph, is discharged into the thoracic duct. The lymph and chyle when discharged into the large veins at the root of the neck mix with the venous blood, and pass through the superior vena cava into the right auricle of the heart.

Although the lymphatic system may be regarded as an adjunct to the venous portion of the circulation, and lymph-like venous blood flows from the periphery to the centre, that is to say from the distant parts of the body to the heart, there are some marked differences between veins and lymphatics. The latter, as a rule, have much thinner and more delicate coats than veins, and are much more richly supplied with valves (fig. 45), which gives to these tubes, when distended by quicksilver or some other form of injection, a distinctly beaded appearance (fig. 46).

Whilst in the venous system the blood is carried from minute twigs to small branches, and then through larger and larger branches to a single large trunk, the arrangement of the veins reminding us of the regular divisions and subdivisions of the trunk and branches of a tree, the lymphatics do not in the interval between their peripheral origin in fine capillaries and their central termination in the thoracic duct regularly increase in size. Here and there they really become smaller and sometimes break up into minute branches which run together again and reform a single trunk. A very marked feature in the lymphatic system is the association with the vessels of the numerous and widely diffused lymph-glands in which the lymph in its course from the tissues to the heart is altered and probably to some extent purified by being brought into close relation with circulating blood, and with accumulations of certain cells or corpuscles constituting what is known as lymphoid tissue. Certain groups of lymph glands, and indeed some single glands, have a definite and constant relation to certain regions or organs of the body. For instance, the lymphatics from the scalp pass through glands seated at the back and upper part of the neck; those from the tongue pass for the most part through glands below the chin and lower jaw, and in front of the neck; those from the throat (pharynx) and tonsil to a short chain of deep-seated glands behind the angle of the lower jaw. The numerous glands arranged in chains and clusters in the armpit receive lymphatics from the breast and from a greater part of the upper limb. The lymphatics of the inner surface of the forearm and the two last fingers pass through a single gland situated on the inner surface of the arm just above the elbow. A small cluster of glands situated over the large femoral vessels just below the fold of the groin receives lymphatics from the leg and thigh.

Although the lymph is not, like the blood in the veins, moved onwards by a propelling force—the so-called *vis a tergo*—transmitted from the heart and arteries and the human being is not like the frog provided with contractile sacs or lymph-hearts—specially adapted to this work, there is a constant onward flow of lymph from the peripheral capillaries to its terminal discharge in the large veins of the neck. This flow is due to several causes, such as the action of muscles, and movements of the different parts, the pressure of tissues on the lymph as it is poured out from the veins, to contractile action of the walls of the lymphatics, which contain involuntary muscular fibres, and, it may be, to some attraction between the inner lining of the tubes and the lymph. The current, when established, must flow onwards towards the heart, as the numerous valves with which the lymphatics are provided are so arranged as to obstruct any flow in the reverse direction.

The study of the lymphatic system forms but a small part of ordinary anatomical teaching, and from their small size,

### LECTURES TO NURSES ON ANATOMY.—*Continued.*

their intricate arrangement, and the necessity for special preparation, both lymph vessels and lymph glands are more or less disregarded in students' *dissections*. In the living subject, however, these organs may acquire the greatest importance, becoming perceptible in many acute affections, some mild, others very severe, and taking part in certain chronic morbid processes that might fairly be regarded as the most prevalent and also the most deadly afflictions of humanity.

The lymphatics in the performance of their function of absorption have no rights of rejection or selection, and so may take up with the lymph any impure or poisonous matter, or any germs of disease introduced either from without or developed within the organism. Whilst the lymphatics as a rule take up what is presented to them of such hurtful matter, the glands make some effort to protect the rest of the body and to act, more or less efficiently, according to the nature of the attack, as filters. Such efforts, however, too often result in destructive inflammation of the friendly gland, or in the development of disease which may extend to neighbouring glands, and sooner or later invade with serious, and too often with fatal, results other organs of the body.

As familiar instances of the reaction of the lymphatic system to influences of injury and disease, I might remind you of the red and inflamed arm with painful and sometimes pus-forming swelling from glandular inflammation near the elbow or in the arm-pit, so often observed after a small poisoned wound of the finger or of the hand; of the enlarged and tubercular glands formed in the neck as the result of infection by the tubercle bacilli, and of the still more severe and formidable glandular enlargement due to extension of cancerous disease from the organs to which in their anatomical relations the affected glands correspond, as, for example, the glands of the arm-pit in cases of breast cancer, and the glands of the neck in cancer of the lip and of the tongue. The grave import of such swellings is indicated by the tendency of modern surgeons in operating for cancer to regard removal of the original disease as a subordinate though essential part of their treatment, and to direct most of their care and skill to a thorough extirpation of all the glands, whether they be appreciably diseased or not, that derive their lymph currents from the organ primarily exposed to the malignant attack.

### Beyond the Seas: Nursing in India.

BY A CORRESPONDENT.

Though the East is the East, and the West is the West, it cannot be denied that during the last 30 years or so there has been steady progress in India towards the establishing of certain social amenities universally regarded as vital for the maintenance of any valid claim to civilisation in its proper sense. Of such obligations one of the most outstanding is the intelligent systematic care of the sick, to which is joined all that is included under preventive medicine. Much has been done towards the discharge of both of these, but it has been abundantly proved that the highest medical skill, the most excellent provisions for treatment, will fall far short of their aim if unsupported by skilled nursing; though it is possible that only those who have actually experienced the difference between treating cases with good nurses in charge, and treating other cases without such assistance, can fully appreciate the difference. In several directions attempts have been made to alleviate the burden of suffering borne by the people of India, and with varying success. The civil population is a decidedly heterogeneous one. In point of quantity, alone, it is enormous, more than three hundred million persons. In point of quality it is composed of very numerous races and of very varying elements. It is surely obvious that for the welfare of the sick amongst these millions there is ample scope for an almost unlimited amount of skilled nursing aid, but it must be admitted that for anything approaching a complete system there is neither the money nor the means of organisation available. The planting, the military, and the missionary communities may be trusted to make their own arrangements, but I may note in passing that the first-named might with great advantage formulate some scheme by which the various centres would each agree to maintain two or more trained nurses to work under the direction of the medical men employed by the planters, preferably in a small home hospital, situated in as favourable a locality as possible. I have often heard complaints from planters as to the need for something of this kind, and I am sure that their doctors would feel only too glad to obtain the assistance afforded by the presence of skilled nurses for their bad cases.

### The Up-Country Nursing Association.

So far as those members of the official and mercantile communities who reside in the *mufâsil* are concerned, the "Up-Country Nursing Association," with which is associated the "Wenlock Nursing Association" in Madras, has been formed with a view to providing, at short notice, the services of highly-trained British nurses to any applicants wherever resident. That there is a great and real necessity for such an association anyone with Indian experience will readily admit. Only those who have gone through it can realise the horror, not to speak of the grave risk to life, of a long and serious illness in an out-of-the-way "station," where, saving the daily visit of a busy civil surgeon (himself liable to be summoned at a moment's notice to other parts of his district) or one of his subordinates, the wretched patient has to carry on the struggle as best he or she may, nursed (*sic*) chiefly, or even entirely, by ignorant servants. Granting that the latter are usually at their best when on the march or attending a sick person, the fact remains that their knowledge of sick nursing is fairly covered by the one word *cunji*, whilst their persons and belongings are as far removed from a condition of asepsis as it is possible to imagine. Of course, friends are very kind and do what they can; but the official population is a busy and largely a shifting one, "in" to-day and "out" in the district to-morrow; whilst ladies with any experience in nursing are not as a rule available when wanted, nor can they leave their own domestic duties. From the patient's point of view, then, the work of this Association is most laudable and worthy of generous support and encouragement. From the nurse's point of view the work is undoubtedly hard, mainly because of the trying climatic conditions and the wearing effects of travel *plus* work in India. There, also, as here, the idea is still too prevalent that a nurse, unlike anyone else, save perhaps a doctor, is so constituted as to be able to do duty continuously day and night. On the other hand, there are compensations of several kinds, not the least of which is that the patients are mostly of a very pleasant class socially, and accustomed to endure without complaint discomforts, and even hardships, especially in the way of food and accommodation, which would appal most persons in this country. For a well-trained nurse about 25 to 30 years of age, and of strong, preferably "wiry," constitution, a period of ten years' nursing in the *mufâsil*, with a holiday of not less than six months in Britain after five years and a month on the hills annually, would prove, I believe, most interesting

### BEYOND THE SEAS.—*Continued.*

and profitable in every way. Further, there is an increasing chance of securing permanent and somewhat easier work either at a hill station or in connection with some public institution, official or otherwise, possibly in a good climate on the plains. But I repeat that a nurse must be physically strong and highly-trained if she is to maintain a successful position against locally-trained and more or less acclimatised nurses, some of whom possess excellent attainments.

### The Large Hospitals.

As to the large hospitals in the Presidency towns, there has been of late years an immense improvement, due partly to the constant pressure exerted by the medical staffs of those hospitals upon the higher administrative authorities, and partly to the appointment of highly-trained and capable nursing heads who have succeeded by unwearied personal effort in raising the standard of Indian nursing to a point never approached in former times. Fifteen years ago, more or less, anything in the way of food, accommodation, or salary was good enough for a nurse, and, as a result, any kind of person, respectability being quite a secondary consideration, was good enough for the "job." Even now, matters are not by any means altogether satisfactory in regard to the treatment of the nursing staffs at the large hospitals; but there is a steady upward tendency, and it is hardly too much to hope that in time the official view of a nurse's legitimate requirements will approximate to that of those who know what nursing in a sweltering climate, year in year out, really means. At one large hospital in the 'eighties there was an incompetent matron and no assistant matron or staff nurses, whilst the "nursing" was limited to the taking of (approximate) temperatures, the administration of doubtfully-compounded medicines, and a very general supervision. Of true nursing there was little by day and less by night, the not unusual equipment of a nurse on night duty being a long chair, a pillow, and a novel! With a few honourable exceptions a nurse could only be relied upon to tell the truth by accident. Since that time a vast improvement has been slowly but steadily effected, and it has been proved that the standard of Indian nursing can at least be approximated to the high British standard, with the greatest benefit to all concerned, and without doing any violence to caste or other scruples on the part of the patients. There is, however, one important fact which strongly differentiates the great Indian from the great British hospitals, namely, the official character of the former. The Governments in India spend enormous sums of money annually in affording medical aid to the native population, whilst, with the exception of Bombay, a hospital built, equipped, and maintained by private charity—excluding missions—is almost unknown. Naturally enough, those who provide the money keep the administration entirely in their own hands. Unfortunately, the higher administrative authorities, with rare exceptions, are unable to recognise the vital importance of a properly-organised nursing staff, well-paid, well-officered, and of sufficient numbers to guarantee that the patients shall be really nursed, and not left to the tender mercies of ward coolies. Further, such a thing as an educated public opinion hardly exists in India, and in any case the only means of giving expression to that which does exist is through the newspapers. It must be admitted that the editors of the latter are always willing to do what can be done. So far, then, as the patients in these great hospitals are concerned, there has been a vast improvement of late years in their treatment and surroundings, but there is still much to be accomplished ere the sick can be said to be properly nursed. As to the nurses, their lot is much improved; but in their case, also, there are many questions demanding careful consideration, not the least difficult of which is the attempt to secure a satisfactory *modus vivendi* in view of the many differences, ethical and social, between many of the nurses locally bred and trained and those who are imported prior or subsequent to training.

### District Hospitals.

Concerning the nursing arrangements for the numerous hospitals situated in the head-quarters town of each district, it must be acknowledged that at many of these hospitals there is no provision for nursing at all; a few ward coolies, male and female, for carrying food and similar duties, representing the nursing staff. At other hospitals, again, there may be one elderly female, the Eastern prototype of Sairey Gamp, conspicuous chiefly for size, indolence, and an almost entire ignorance of her duties. In a few—a very few—hospitals there are one or more nurses who may be fairly competent, and occasionally—very occasionally—one comes across a nurse who is really efficient and does her work conscientiously in spite of many difficulties and discouragements. The hospitals are in charge of the civil or district surgeons who, for the most part, are officers of the Indian Medical Service. These officers, as a whole, must be acquitted of blame in the matter, for it is well known that by far the greater number themselves deplore the existing conditions, and would alter them for the better to-morrow, were it in their power to do so.

### The Question of Funds.

The invariable cry is "no funds!" It must be admitted that there is much truth in this, since the population of India is for the most part a very poverty-stricken one. There is, however, in every town of any size quite a sufficient number of comparatively wealthy individuals well able to subscribe handsomely towards the upkeep of the hospital, but who never by any chance do so. Many of these people are charitable enough in their own way and subscribe largely to temple funds, rest-houses for pilgrims, etc., but are deaf to all ordinary appeals for the local hospital. The result is that the latter institution is starved, and shift has to be made with a wretched building, a defective equipment, and no provision, certainly no proper provision, for nursing. Admittedly, there are many difficulties to be got over, but I consider that the time has come when the problem of bringing these hospitals up to date should be boldly attacked, especially with a view to the provision of well-trained nurses. For the comfort of the nurses proper arrangements must be made, suitable quarters constructed or allotted, and at least a living wage be paid. If such be done there will be no difficulty in finding women who have been trained under conditions favourable to the development of self-reliance and good principles, to undertake the duties and carry them out satisfactorily. On no account should the cry of "no funds" be regarded as a hopeless barrier. If anyone had suggested the idea of the Dufferin hospitals or of providing skilled midwives to the women of India a few years prior to the successful appeals made for these objects, he or she would have been regarded at the best as a visionary, at the worst as a confirmed lunatic.

### Improvements Practicable.

The improvements outlined can be, and should be, made, but they will not be accomplished without great labour, patient consideration of ways and means, and the arousing of public interest by every legitimate method. Lady Curzon has already earned the gratitude of the Indian people by her efforts to secure the safety of the mothers of the Empire in childbirth, and she has been ably seconded by the women of India, European and Native. She will earn the lasting gratitude of millions more if she will further devote her time and energies to arousing the interest and co-operation of her sisters in India towards the creation of an efficient Civil Nursing Service for the Presidency and District civil hospitals throughout the Indian Empire; a task toward the completion of which the assistance of the women of Great Britain may well be invited, and, as I believe, not in vain.

### Death of a Crimean Nurse.

### ANNE EYRE HELY, R.R.C.

One more of Miss Florence Nightingale's staff of nurses in the Crimea has gone to her rest in the person of Mrs. Anne Eyre Hely, who died last week at the Almshouses, Ravenstone, Ashby-de-la-Zouch. Mrs. Hely, whose maiden name was Bradshaw, was born at Ravenstone, and nothing eventful is recorded in her life for many years. Miss Bradshaw married a country doctor, but was early left a widow and thus thrown upon her own resources. In the year 1854 she left England to join the brave band of women working under Miss Florence Nightingale in the Crimea. Mrs. Hely had at one time under her charge 150 patients, most of them suffering from gunshot wounds or terrible frost bites. She did not return to England until after the war was over and the soldiers were convalescent and also coming home. Upon her arrival in England she was selected to nurse Miss Nightingale herself for some months, and afterwards was chosen to nurse the late Duchess of Kent in her fatal illness. But the Duchess died just as she was going to commence her duties. Feeling after this time the necessity of a change in her manner of life, Mrs. Hely became in turn housekeeper in the households of the Earl of Enniskillen and the Marquis of Zetland, in both of which positions she was much respected and esteemed. Advancing years making a permanent rest desirable, her thoughts were turned to her native village, where she found a peaceful happy home in the almshouses founded many years ago by a generous and philanthropic Leicestershire man. Her case was brought before the late Queen by the chaplain of the almshouses, Dr. Barber, and Her Gracious Majesty at once bestowed upon Mrs. Hely the "Order of the Royal Red Cross." This Order was founded in 1883, and, at the time Mrs. Hely received it, there were only 75 persons who were the proud and happy owners of it. Her health was then too weak to allow her to undertake the journey to Windsor for the proper investiture, so the Queen, with that kind sympathy and consideration which never failed throughout her long reign, permitted the ceremony to take place at Ravenstone. At an enthusiastic meeting of friends from the neighbourhood the Order was pinned to Mrs. Hely's breast by Lady Cave Browne Cave, a lady whose kindness and sympathy is well known. This was in the year 1897, and now, in 1902, at the age of 83, Mrs. Hely has passed away, leaving an example to all women, and especially to nurses.

### Nursing in the Concentration Camps at Krugersdorp.

### BY AN ENGLISH SISTER.

We left Southampton on December 20th, and after a pleasant voyage landed at Capetown on Sunday, January 12th. After waiting about in Capetown for permits, we commenced our up-country journey on the evening of the 14th. We travelled by night and day until we reached Bloemfontein, where our train was put into a siding until 4 A.M. The journey up was most interesting. The guards were very good in coming round and telling us when we were nearing any place of note. We reached Johannesburg at 7 P.M. on Friday the 17th, and, after reporting ourselves to the military staff officer and obtaining permits to leave the station, we went into the town and stayed at the hotel for the night, and left Johannesburg again at 7 A.M. for Krugersdorp. The first of our party had left us at Vereeniging on the Transvaal frontier, and nine others left us at Ellandsfontein, where they changed for various parts of the Transvaal. We arrived there about 9.30 A.M. on Saturday, January 18th. We were met by the superintendent of the camp, and were taken to the hotel, as our tents were not pitched. On Monday, the 20th, exactly a month after leaving England, we were driven up to the camp, and slept in tents for the first time in our lives.

### The Sisters' Duties.

There are 4,000 refugees in this camp. The nursing staff consists of certificated matron, relief matron, two trained sisters, and three nurses, in addition to which there are about 18 Dutch probationers, whose duty it is to go round and visit every tent early in the morning, find out any new case of sickness, distribute medical comforts to those already on the list, also see that the tents are clean and well ventilated. They then come with their reports to the sisters, who first have an hour in the out-patients' surgery, where the medical officers see slight cases. The sisters next visit the cases in camp along with the doctor, receive his instructions, go round again with the probationers and teach them as much as possible of home nursing. The girls are very willing pupils and are becoming very helpful. Our morning rounds are usually over by 12.30, we have lunch at 1, then off duty until 4.30, when we have a cup of tea and go back to camp to visit our worst cases. We have dinner at 6 P.M. and are then off duty for the rest of the day, unless we have any very bad case, which, of course, we see again before bedtime. When we first came we were in camp from morning to night; but now it is much lighter, not only on account of the improved health of the camp, but also because of the greater help which we receive from the Dutch girls.

### The Other Camps.

The whole camp is divided into five smaller camps or wards and numbered Camp I., Camp II., etc., each sister or nurse is responsible for a camp, with a certain number of probationers under her. The average number of tents for each probationer is about eighty. There is a perfect feeling of goodfellowship throughout the camp, and the majority of the people have unbounded confidence in both the doctors and sisters. All enteric cases are removed to hospital at once, and everyone from the same tent has to go to the "contact camp" which is placed a little distance away from the general camp. The tent is taken down and fumigated and the ground dug up and disinfected. The Quarantine Camp is called by the probationers "The Turpentine Camp." All refugees coming from other camps must first stay for 14 days in this camp before mixing with the people, to prevent any infectious disease being imported. During the last three months the camp has been extended east and west, giving greater distance between tents, which has no doubt a great deal to do with the improved condition of the people. During March there were only five deaths in the whole camp, including hospital, and during April only three deaths. The hospital contains 50 beds. The nursing staff consists of trained matron, four trained sisters, and about 20 Dutch probationers.

### The Various Institutions.

There is an orphanage with 53 children with a trained nurse in charge, and two of the Dutch girls, who have been trained as camp nurses, as assistants. There are 1,100 children attending the schools, an English schoolmaster with a staff of English teachers, and also Dutch assistants. The children seem to like attending very much, and are in great trouble if they are kept away by sickness. They are learning to speak English very nicely. It is not all work; we also have our recreation. There is a "Staff Tennis Club," with a very good court. Once a week is an open day when any member may invite friends. Tea is provided in a marquee adjoining the tennis court, the ladies taking it in turn to preside. There is a large recreation hall, with ping-pong tables, cards, library, chess, etc. The superintendent allows his piano to be used. The large marquee which was used before the war by Mr. Kruger for service here once a year, is now employed as a church and is well attended. We have no peal of bells, but a boy walks about the camp ringing a large hand-bell like a town crier for a quarter of an hour before each service. There are three services on Sunday and one on Wednesday afternoon, some of these are conducted by the minister from the Dutch Reformed Church in the "Dorp," and the remainder by a lay preacher resident in camp. It is a strange sight to see the people on their way to church on Sunday morning, walking down "Oxford Street," a broad road through the centre of the camp, each carrying his or her own chair. We have just had one of our "camp babies" baptised. Hermina Catrina Bertha De-la-Rey was the name given. Her father is cousin to the general.

### The Support of Helpless Patients in Bed.

EXAMINATION QUESTIONS FOR NURSES.

THE question was as follows:—"What means would you employ to overcome the continual 'slipping down' in bed of an absolutely helpless patient who is ordered to remain in a semi-recumbent position, or is compelled to be sitting up in bed?"

### FIRST PRIZE.

I would roll a pillow into a draw-sheet, leaving sufficient quantity of the sheet on either side of the pillow to make a loophole. The end of the draw-sheet on both I would fasten down severally and securely with safety pins, as near to the pillow as possible, and so form the loop through which I would thread a good strong bandage. I would then arrange the pillow under the legs of patients well up above the knees, and secure the position of the same by fastening through the ends of bandages to the head of bedstead on either side of patient. In a cottage where no couch is available, and the patient is in a chronic state of absolute helplessness, but allowed to sit out of bed in an upright position, I would place the pillow (hitherto described) to the front part of the seat of an armchair, leaving sufficient space for the patient to sit comfortably between the pillow and back of chair, then take the bandage under the arms of chair and fasten securely at the back. Place a block under each foreleg of the chair: these blocks lend a semi-reclining position to the chair, and not only help to keep the patient from slipping, but do, I believe, give a little more ease and comfort to the patient. An ordinary chair added will then make the length of couch you require.—"HORWOOD."

### SECOND PRIZE.

If patient is to be in semi-recumbent position, place bed-rest or upturned chair well covered with pillows behind patient's shoulders, two pillows to come well out on either side for rest and protection of patient's arms. Fix patient up comfortably, then get a firm pillow and place under buttocks. Attach to either end of pillow strong tape or strap, and tie these firmly to head of bed. This can be done, without interfering with patient's comfort, by tying low down on bedstead. A further help may be afforded by raising the foot of bed slightly.—"OMEGA."

### THEIR MERITS AND SHORTCOMINGS.

"Horwood" gains the first prize on two accounts. She recognises, as "Omega," the second prize-winner does, the importance of giving a counter-tendency *upwards* just at the heaviest part of the body, and also because she shows herself a woman of resource in describing what she would do in a cottage where appliances are few, and where, in spite of doctor's orders and in the absence of the district nurse, the patient is usually taken out of bed and propped, more or less precariously, in a slippery Windsor chair. In carrying out "Horwood's" idea be careful to fix the chair in which the invalid sits beyond any possibility of tilting back.

"Omega's" answer is succinct and good; it is a pity not to have been more careful in the composition. The noun *patient* occurs five times in 94 words. Neither prizewinner mentions the relief obtained by making some arrangement across the bed in front, on, or against which, the sufferer can lean, when breathing is easier in that position.

### HONOURABLE MENTION.

This is awarded to "Coronation," "Gypsy," and "Magdalene." Two papers have been sent with the pseudonym "Gypsy." To avoid disappointment I must mention that the successful candidate comes from Chesterfield.

"Magdalene's" paper is good in substance but careless in execution. Like "Omega," she clings tenaciously to the word *patient*, and introduces it 17 times. You may think this a matter of small importance, but reflection will show the contrary. If a nurse hopes to attain a position where it is necessary to instruct others, it is of immense advantage that she should know how to clothe her information in simple, telling, and impressive language, and not weary her hearers with confusing sentences.

### DEFECTS IN OTHER PAPERS.

I consider it a serious want of observation not to realise that no amount of raising the foot of the bed, or any other arrangement for the feet, will materially overcome the slipping-down tendency. Look upon the matter as a question of engineering. Wherever the weight is greatest, *there* will the *sinking down* occur, and the only remedy is to apply a counter-traction upwards, to be accomplished by a continual slight pull towards the head of the bed by the pillow under the buttocks attached to the iron frame of the bedstead. It is comfortable to a helpless person also as giving absolute support. Some candidates speak of the pillow under the buttocks, but say its encasing draw-sheet should be tucked into the mattress—a most feeble and futile plan. Pillows under the knees are a great comfort, and something against the feet is a useful auxiliary, but nothing answers like the *upward pull.*

"Paddie," who has been successful more than once, is this month disappointing. She pins her faith on a webbing support round the shoulders—a pernicious contrivance that, however well padded, would soon gall, and in dropsical cases have very serious results.

"Skylark" enters into all sorts of irrelevant matter. What bearing have temperature and ventilation on the question given?

"Daisy" also affords much undesired information on outside matters, but barely touches the subject she is asked to consider.

### QUESTION FOR JUNE.

How would you treat (so far as a nurse's province extends) a severe case of rheumatic fever? N.B.—You are not asked *anything* with regard to drugs and medicinal remedies—which treatment rests with the doctor alone—but simply as to personal attentions, diet, etc. The question of diet is also naturally one for the medical man's decision; but inasmuch as pressure on his time, distance from his patients, and other causes, especially in outlying districts, often preclude his giving his attention to the matter, he frequently trusts the nurse working under him to settle what she thinks best as circumstances arise. It is therefore well that she should be prepared to act for the patient's benefit in this respect should the doctor wish it.

THE EXAMINER.

### Wants and Workers.

DISTRICT NURSE, 21 Chester Street, Saltney, Chester would be very grateful for parcel of old linen for poor woman.

WOULD any nurse like a portable Turkish hot-air and vapour bath (Hawksley's) no longer required? Also wooden wool-winder, 22½ inches high, and some white cotton stockings. Apply M. D. B., 11 Queen's Gardens, Bayswater. Pay carriage.

### Novelties for Nurses.

BY OUR SHOPPING CORRESPONDENT.

### BICYCLES.

IF any of my readers are thinking of buying a new bicycle I should recommend them to write for a catalogue to Messrs. Edward O'Brien, Coventry. He has a special make of machine which he recommends for ladies, and as I see in his list of testimonials a number of names of nurses, I think I am safe in saying that his offer is worth considering. The bicycle he most strongly recommends is a "Coventry Challenge," guaranteed for two or four years, according to make and price. Special terms are offered to nurses.

### "The Hospital" Convalescent Fund.

WE have to acknowledge the receipt of 20s. from a "Grateful Nurse" to THE HOSPITAL Convalescent Fund.

### English Nursing in Rome.

BY A TRAINED NURSE.

THE Anglo-American Nursing Home in Rome is such a comparatively new institution that probably very few nurses know anything about it, and as one who was attached to the staff there in its first year, what I can tell may be of interest to them. English-speaking travellers and tourists who had the misfortune to be overtaken with illness while passing through or visiting Italy, suffered so much from lack of necessary care, partly from ignorance of the language of the nuns who are the only nursing community in Italy, and partly from the want of trained knowledge in these, that the English and American residents in Rome at length most generously and warm-heartedly took up the cause of their unfortunate fellow-countrymen, and set on foot a scheme for providing them with nurses of English speech and training, and a nursing home conducted by Englishwomen and carried on in the same way as such institutions are in England, so that the discomforts and expense of enduring an illness in an hotel in a foreign land might be obviated.

THE START.

The scheme met with the greatest opposition on the part of the Roman Catholics in Rome, who were naturally anxious to keep the nursing elements in their own hands, and in spite of all the efforts of the English and American residents and medical men, it was found almost impossible to obtain a house for the purpose. A matron and several nurses who had gone out had to remain in lodgings, but their services were found so acceptable that the committee felt encouraged to continue their efforts, and the next year succeeded in getting a villa about a mile outside the walls of Rome, but in immediate communication with the city by an excellent service of electric trams, and of course by telephone with all the medical men, hotels, and chief shops. So in October 1900 the matron and two nurses went out to get the house ready for the reception of patients, and for the nursing staff who were to follow about the end of November, when the Roman season begins. I was one of the two nurses, and never shall I forget our arrival in Rome at midnight, after the long wearisome journey from England. We were all three so tired, and oh! the feeling of relief when we found that one of the members of the committee had most thoughtfully sent his carriage for us and his valet to see to our luggage and spare us all the fuss and worry of making ourselves understood. But alas! the valet brought letters, and in the dim light as we drove along the matron deciphered the unwelcome fact that a nurse was wanted immediately for a case in an hotel, and that the man who had been engaged as porter and general factotum at the house, and who had been acting as caretaker until we arrived, had died the previous day, and his widow, who was the cook, was incapacitated with grief. The good friend who had sent to meet us had himself engaged a German maid and sent her in that day, and had seen that our beds and some food were ready, and we felt that all else must wait until we had had a night's rest, for neither Nurse B. nor I were fit to go straight to a case. However, next morning at 10 o'clock, away she went, and the matron and I set to work to arrange the house.

THE HOME.

The Home is a nice square-looking building, standing well back from the road in its own garden, which makes a cheerful promenade for convalescing patients. The entrance hall is most inviting with its short flight of wide steps and its marble pillars; on the left-hand side is the matron's office and sitting-room, with her bedroom leading out of it; that again communicates with a large room at the back, which is furnished at one end as the nurses' sitting-room and the other half is the dining-room; adjoining this is the store-room and porter's room; on the opposite side is the maids' room. In all Italian houses each room communicates with the one on either side of it, as well as having its own entrance from the hall or landing. The floors, of course, are all tessellated, and the wide staircase from basement to roof is of marble, cool enough in summer, but very uncomfortably cold in winter, and as trying to one's feet as the floors and stairs of an English hospital. On the first floor are the five rooms for patients, with bath-room, etc., all opening out of a small square landing which can be quite shut off from the outside landing and staircase, and in this are cupboards for the necessary appliances and small stores, and here also the nurse or nurses sit at night or when not required in any patient's room, and, as all the doors open into it, a very good watch can be kept on all at once. On the floor above this are the nurses' rooms and a room which can be used for temporary isolation if anything infectious should occur.

THE WORK.

The staff consists of about eight nurses, two of whom are engaged for the work in the home and the other six are engaged to take private cases at hotels or in private houses, or a "special" case in the home if such is required; also if there is a pressure of work in the home a disengaged private nurse generally is ready to relieve and help the possibly overtaxed home nurse. Occasionally it happened that for a day or two the work was very light, and then was the time to make the most of the opportunity and see as much as possible of the wonders of "the Eternal City." The roof was a very popular place of resort for a few minutes' recreation; the view from it is so lovely, right across the fascinating Campagna to the snow-capped heights of the Appenines and Alban hills, and then their colouring with the rays of the rising or setting sun is a picture that is indelibly impressed on one's mental retina. Patients are admitted as in English hospitals quite irrespective of their religious views, and two beds have been endowed for the use of patients whose circumstances prevent them from contributing any fee however small, whereas other patients pay from 15 to 20 francs per day. Last year the home was closed from July to October, but I believe that arrangements are being considered for keeping it open all the year. With regard to the nature of the illnesses which were nursed in the home last year, I will quote from the report. "Twenty-four have been cases of acute disease such as bronchitis, pneumonia, enteritis, etc.; nineteen of chronic diseases, while five have been surgical cases, in four of which operation was necessary." As this is the only institution of the kind in Italy, the private nurses were a demand in other towns also. One nurse went to Palermo, a second to Florence, a third was wanted in Venice, and of course the nursing in the hotels is much the same as in any foreign town.

### A Hospital Ship for Fishermen.

THE Bishop of London on Tuesday morning dedicated the second of three hospital ships for the fishermen in the North Sea, built by the Royal National Mission to Deep Sea Fishermen. The vessel, which was lying at Billingsgate Wharf, was gaily decorated for the occasion, and a number of people, among whom were several nurses, attended the short dedicatory service, while many more lined the wharf, and several barges came up alongside, their inmates evidently much enjoying the singing of the hymns. The steamer is named the *Queen Alexandra*, and at the close of his address the Bishop took the opportunity of announcing a fact which was not previously known, *i.e.*, that the Queen, anxious that no one should be left out of the Coronation festivities, had asked him to see that 10,000 maids-of-all-work in London were provided with tea and a medal stamped with the heads of the King and Queen. A Coronation Hymn by Louisa White was sung, and the service closed with a verse of the National Anthem. The visitors afterwards inspected the ship, which, though on a small scale, is quite up to date, and is fitted with the X-rays. One surgeon and two or more mission workers will sail for the North Sea during the course of this week, carrying, besides medical comforts, literature of a wholesome kind. A similar vessel to the *Queen Alexandra* is on the stocks. The cost of each is about £12,000.

### The Nurses of Friedenheim Hospital.

OPENING OF A NEW HOME.

A large number of persons assembled at Friedenheim Hospital last Saturday afternoon for the opening of the new Home for Nurses. The rain, which fell both before and after the ceremony, ceased for a couple of hours, but the pleasure of the guests would have been greatly enhanced had it been possible to sit under the trees in the lovely garden, where lilacs and laburnums were in full blossom.

A marquee had been erected on the lawn, and Her Royal Highness Princess Christian, who was accompanied by her daughter, Princess Victoria of Schleswig-Holstein, was received by the reception committee, and conducted to the platform, which was beautifully decorated with palms. Two little children, the Hon. Victoria Bruce and Master Alastair Davidson, presented bouquets of red and white roses and carnations to the royal ladies, and then Mr. J. H. Tritton, chairman and hon. treasurer, welcomed the Princess as patron of the hospital, on behalf of the council.

This was not the first occasion, Mr. Tritton said, on which the Princess had honoured the institution with her presence, for at a time when it had been but recently opened by the late Duchess of Teck, Princess Christian paid a visit to it, and named the Helena Ward after herself and the Princesses Victoria and Louise. Mr. Tritton went on to give some details of the history of Friedenheim—how it was opened in 1885 in a small house at Mildmay Park, and removed to Swiss Cottage in 1892, and how nearly 1,200 patients had been received, while over 800 had passed away, tenderly nursed and cared for to the end.

"While much care has been bestowed on the patients," Mr. Tritton continued, "the accommodation for the nurses—whose duty and privilege it is to watch over them—has been totally inadequate. Owing to want of space, some of the nurses have been lodged in premises only adapted for the purpose, and quite unsuitable for a permanent arrangement. Taking these facts into consideration, and also the inadvisability of allowing those who nurse advanced cases of cancer and consumption to spend their leisure and sleep in the same atmosphere, and also, indeed, owing to the strong representations of Miss Davidson, the council felt it to be an imperative necessity to erect the building which your Royal Highness has come to open. In this building each nurse will have a separate bedroom, and there is a bright sitting-room for their use when off duty."

Referring to the finances, Mr. Tritton said that a sum of £3,500 was required to complete the building, and to efficiently adapt for the use of the hospital the rooms vacated by the nurses, while the new Home itself would entail an increased annual expenditure. He did not doubt that Her Royal Highness's presence, showing her approval of the proposed extension, would serve as an incentive to many to help forward this good work.

The hymn "O Praise our God to-day" was sung, prayer was offered by the Rev. S. A. Selwyn, vicar of Holy Trinity, Hampstead, and then the Earl of Aberdeen, Sir J. Crichton Browne, F.R.S., and Dr. Schofield having addressed the meeting, the Princess said, "I am very pleased to declare the new Home open, in which I hope the nurses will find rest and recreation from their work."

This concluded the business, and, after tea, the visitors went over the Home, and were delighted with the arrangements for the nurses' comfort. All the rooms are simply but prettily furnished; those facing north are coloured a cheerful pink, while the rooms that will get the most sunshine are of a cool blue tone. The sitting-room and passages are coloured in a pale creamy primrose shade, and the wide staircase gives an artistic finish to the whole.

### Appointments.

[No charge is made for announcements under this head, and we are always glad to receive, and publish, appointments. But it is essential that in all cases the school of training should be given.]

Bridgend Cottage Hospital.—Miss Annie Tanner has been appointed matron. She was trained at the Royal Infirmary, Bradford, where she was gold medallist for 1897–8. Since then she has been charge nurse at the Rochdale Infirmary, sister of the Women's Medical wards at Wolverhampton General Hospital, and matron of the Memorial Hospital, Almondsbury, Bristol.

Fulham Infirmary.—Miss Olive Pollett and Miss Mary de Angelis Willett have been appointed sisters. Miss Pollett was trained at Poplar Hospital, where she has since been staff nurse. She has also been nurse at the Sussex County Hospital, Brighton. Miss Willett was trained at the Royal Infirmary, Liverpool, and has since been nurse at St. Mary's Hospital, Manchester.

Infirmary and Dispensary, Bolton.—Miss Dryden has been appointed night sister. She was trained at the Children's Hospital, Birmingham, and St. George's Hospital, London, and has since held the position of sister at the Grove Hospital, Tooting.

Monkwearmouth and Southwick Hospital, Sunderland.—Miss A. I. Horrocks has been appointed charge nurse. She was trained at Monsall Fever Hospital, Manchester and North Stafford Infirmary and Eye Hospital, Stoke-on-Trent, and has since been sister at Fulham Infirmary.

New General Hospital, Stobhill, Glasgow.—Miss Marguerite Wright has been appointed matron. She has been lady superintendent of the Town Hospital, Glasgow.

Passmore Edwards Cottage Hospital, Wood Green Miss S. Leach has been appointed matron. She was trained at the Royal Bucks Hospital, Aylesbury, and has since been staff nurse at the Jaffray Hospital, Birmingham, sister at Grantham Hospital, and charge nurse at the Hospital for Women, Derby.

Royal Infirmary, Sheffield.—Miss Katharine Jones has been appointed assistant matron. She was trained for three years at Mill Road Infirmary, Liverpool, and was afterwards for four years theatre sister, and for two and a half years night superintendent at the same institution.

Salop Infirmary, Shrewsbury.—Miss E. J. Brown, Miss E. A. Rees, and Miss Copeland Davies have been appointed sisters. Miss Brown was trained at Hartshill Infirmary, Stoke-on-Trent. Miss Rees was trained at Swansea Infirmary, where she was subsequently sister. She has also been sister at Lewisham Infirmary and the Jaffray Hospital Birmingham. Miss Davies was trained at the Royal Infirmary, Liverpool.

Victoria Hospital, Folkestone.—Miss M. E. Morrison has been appointed sister. She was trained at Arbroath Hospital, N.B., and has since been staff nurse at the Sick Children's Hospital, Edinburgh, and sister at Grantham Hospital.

### Presentations.

Palmer Memorial Hospital, Jarrow-on-Tyne.—Miss Storey, on leaving the Palmer Memorial Hospital to take up her post as matron of the Montgomery Infirmary, Newtown was presented by the house surgeon, on behalf of the medical and nursing staff and secretary, with a handsome silver teapot.

Reading Union.—Following on the presentation to Miss S. Pinington, by Lady Wantage, on behalf of the Workhouse Infirmary Nursing Association, the former was the recipient last week of another handsome present in the shape of a massive marble eight-day timepiece, bearing the following inscription: "Presented to Miss S. Pinington by Guardians, past and present, of the Reading Union as a mark of esteem after 21 years' service as Superintendent Nurse, 1880–1901."

### Everybody's Opinion.

[Correspondence on all subjects is invited, but we cannot in any way be responsible for the opinions expressed by our correspondents. No communication can be entertained if the name and address of the correspondent are not given as a guarantee of good faith, but not necessarily for publication. All correspondents should write on one side of the paper only.]

### NURSES' UNIFORM.

"Semper Fidelis" writes: I fully endorse all Sister Marion says about the protection of uniform, but I think that the poor maternity nurse of six months' training should also be allowed to wear out-door uniform, for if we waited to change into private clothes every time we went out for a short walk, few of us would get out at all. Besides, who of us could afford to carry all round the country private hats, dresses, etc.? I think that each training school ought to have a distinct badge, then the impostors would soon be detected.

### THE TESTIMONIAL OF A MATRON.

"Placidity" writes from beyond the seas: In reference to a letter signed "Discussion" in The Hospital of May 3rd, in which the writer considers a member of a hospital staff leaving at her own wish hardly correct, because the matron refused to give her a testimonial, there is another side to the question, as the following particulars will show: A probationer is entertained for one year's training, being housed, fed and paid during that year, on condition that she serve the hospital for two succeeding years at an increased salary (£30 per annum). Before completion of her agreement relatives offer to obtain for her a situation (salary £50 per annum), for which she leaves the hospital service. Is the matron justified in giving the nurse a testimonial under these circumstances? Is it to the best interests of the nurse to leave the hospital for temporary employment before the expiry of her three years' agreement?

### NURSES OF THE MENTALLY AFFLICTED.

"E. S." writes: I shall be glad if you will kindly allow me through the medium of The Hospital to mention a subject which I consider requires attention, and that is the long hours and little opportunity of relaxation and change afforded to those who have the mentally afflicted in their charge in our workhouses—I suppose one of the most, if not the most, trying engagements that a nurse can undertake. I would ask for them shorter daily hours if that can be, three weeks' holiday once a year instead of the fortnight at present allowed, and a Saturday to Monday holiday once in three months in addition to the half-holiday which they now have one Sunday in three. I think this would be good not only for nurses, but also for patients, as they are a class who naturally require a great deal of patience which it is difficult to observe when a nurse is overwrought and over-tired. May I also suggest that persons in these positions should be nurses, not untrained and incompetent people.

### THE FOOD QUESTION.

"M. A. B." writes: Will you kindly let me draw the attention of some of your readers to the nurses' food question. One looks upon a nurse as a lady, educated, and refined, and yet the food, and more especially the cooking, at some hospitals is far from what it ought to be. Take, for instance, breakfast, which twice a week consists of half a bloater, bread and butter, and stewed tea from an urn. A nurse has to commence 13 hours' duty on that. Then comes dinner, perhaps pork which is scarcely warmed through, onions half cooked, and potatoes which look as though they were suffering from yellow jaundice. Then comes boiled rice and a few stale prunes. Tea consists every day of bread and butter; supper, cold meat, bread and water. The officials fare very differently, but the poor hard-working assistant nurses have to put up with such scanty allowance. I will not say that the food is exactly bad, but if the cooking was as it ought to be, surely there would not be so many resignations. If the work in the kitchen were done half as well as that in the wards there would be no room for complaints.

### "NURSES' UNIFORM AND SUPPLEMENTARY TRAINING."

"N. M. C." writes: Kindly permit me to say a few words on the above subject. I followed with much interest Nurse Marion's letter of last week and quite endorse her opinion on the wearing of uniform, having been more than disgusted myself with regard to the nursemaids in town. I would like to suggest to those now agitating the restriction of uniform, that only "certified nurses be permitted to adopt it." No one connected with any other profession is considered a "professional" unless examinations have been passed and certificates obtained. Also in my humble opinion it would be well not to confine the wearing of uniform to three years' trained nurses only, but to permit those who are certified in any branch of the profession to have the privilege of it, as, for instance, those having obtained the L.O.S., the Psychological, or Fever Certificate from a large training school. Many have not a three years' certificate, but have gained one in some particular branch after heavy work and study, and have also the experience of years. It would seem hard, indeed, to force them to lay aside their uniform which no doubt many are as proud of as a soldier is of his regimentals.

### THE ATTACK ON TRAINED NURSES.

"Faith Lynton" writes: Apropos of Miss Johnston's articles in the *Nineteenth Century*, I take the opportunity of relating a little incident which shows how patients are able to appreciate the nursing and treatment which they receive in hospitals. I was working on night duty in a surgical ward in one of the large London hospitals, and amongst my patients had a coalman who several weeks previous to his entrance had punctured his thumb, and on the advice of some "old wives" had tried treating the wound himself. The consequence was the poor fellow was admitted one night, suffering from blood poisoning and with a temperature of 103°. I instantly put him to bed, rolled him in a warm blanket and washed him down as well as I could, although the black was pretty nearly ingrained. However, by the time the house surgeon made his appearance "No. 3" looked fairly comfortable in a warm red flannel jacket. The hand and arm were carefully examined, and then we wheeled the bed into the adjoining "bathroom" where the house surgeon made several large incisions, swathed the limb in cyanide and wool, and put him on four-hourly hot-water dressings. The patient was in a very weak condition on his entrance, and for the next few days we almost despaired of his life. It was, in fact, only by constant "feeding-up" and great care that three weeks later he rose from his bed with his hand still stiff, but quite free from pain or swelling. Ten days later he bade farewell to us altogether—a strong, hale, hearty, north countryman, who could not speak too well of hospitals and nurses, and for the whole treatment which he had received. We, for our part, missed him sorely, for at first he had required all our attention and care and the most gentle nursing, while, latterly, he was an immense help in the ward, and his happy face and northern dialect cheered, not only the surgeons, but the nurses also, who were always ready to listen to his anecdotes, when time permitted. A few months passed away and No. 3 bed had been filled by several patients. I was hurrying out one morning for a blow of fresh air before retiring for the day's rest, and was about to cross a crowded thorougfare when a heavily-laden coal-cart was abruptly pulled up, and the next minute a much begrimed looking man rushed up to me with both hands extended, "Good morning, nurse!" he said, "I'm old '3' that was that ill" (and then I remembered the northern accent and face of our old patient), "why you be our night nurse who was rare good to me. See my hand—it is all here—flesh and bone and all. And the doctor even said as it would have to come off, and so it would have were it not for all you nurses. Why, God bless my soul. I can never thank you all enough! and I tell all my comrades of the kindness and gentleness I received in that there hospital over yonder. That's my mission it be!" A command from a policeman for the cart to "move on" brought this dialogue to an end. The man pulled his forelock and scrambled on to his seat, and in answer to the drivers around, who were grumbling at the delay, he only uttered aloud his thanks and praises for hospitals and nurses.

### Echoes from the Outside World.

### The Thanksgiving for Peace.

On Sunday, everywhere throughout the country, and in all our possessions beyond the seas, services of thanksgiving for the blessing of peace were held in the churches and chapels. In the morning the King and Queen attended St. Paul's Cathedral accompanied by Princess Victoria and Prince and Princess Charles of Denmark. They drove without escort in an open carriage drawn by four horses, and simply preceded by a couple of outriders, clad in scarlet, from Buckingham Palace, by way of Pall Mall and the Strand to Temple Bar where they were received by the Lord Mayor and Sheriffs in State. The former handed over to the King his sign of power, the pearl-encrusted sword, which dates back to the time of Queen Elizabeth, and bade the Sovereign welcome to the City. After a moment's pause the carriage proceeded to the Cathedral, where already there had assembled the Prince and Princess of Wales, the Duke and Duchess of Connaught and their children, Princess Christian and her two daughters, Princess Beatrice and her children, the Duke and Duchess of Argyll, the Duke of Cambridge, Prince Francis of Teck, Lord Roberts, General Buller, etc. The service, which lasted about an hour, was most impressive, and it was remarked how heartily both the King and the Queen joined in the singing of the jubilant hymns. The Bishop of London preached, and during the quieter portions of the service a white dove—probably one of the Cathedral pigeons inadvertently imprisoned—was seen hovering aloft or flying from pillar to pillar.

### The Boer Surrender.

A translation is published of the document issued by the Boer leaders explaining their reason for signing the terms of peace. They said that the conditions showed no hope of winning independence. The country was being devastated, and subsistence for man was vanishing. These were also influenced by the presence of their families in the concentration camps, in which there was sickness. They could not retain thousands of prisoners as the enemy could. Their fewness in the fight against overwhelming numbers of British gave no hope of gaining victory. In spite of their sacrifices they had confidence that by accepting the terms their situation would be so improved that the people might advance to the full enjoyment of the privileges they were entitled to hope for. They expected that an amnesty would be granted to rebels. Wholesale surrenders, numbering many thousands, have been made by the Boers since peace was signed, and a very large number of rifles have been handed in. Conciliatory speeches have also been made by the Boer leaders. On Saturday General Elliott told the burghers at Vredfort Road that the King had telegraphed from England expressing his satisfaction at the termination of hostilities and eulogising the Boers as well as the British forces. The General having added that his Majesty hoped that the burghers would soon be on their farms and would enjoy prosperity and happiness in the future, the burghers gave three hearty cheers for the King.

### War Honours.

The King has conferred the dignity of a viscounty upon Lord Kitchener, and has approved of his promotion to the rank of general. Parliament has also voted him a grant of £50,000. This is half the amount which was voted to Lord Roberts, but the explanation of the smaller sum now awarded is that Lord Kitchener, besides having received a gift of £30,000 after the Soudan War, is still at the height of his career as a soldier, whereas Lord Roberts is advanced in years, and was entitled to exceptional treatment in virtue of his rank as field-marshal.

### Coronation Dresses.

The dresses made in India, under Lady Curzon's orders, for Queen Alexandra have given her Majesty great satisfaction, and she has specially communicated her pleasure at their beauty to Lady Curzon. These dresses were specially ordered so that they might be worn at the Coronation fêtes when so many of the King's Indian subjects will be present. The first instalment of dresses for the Coronation festivities has been sent home to the Princess of Wales, and the dressmaker who was responsible for these confections was not only an Englishwoman herself, but the Princess had stipulated that the material used should be of British manufacture, and that those who were employed in the actual making should all be Englishwomen. One especially dainty summer gown of white muslin is painted in a design of pale blue forget-me-nots, and is trimmed with embroidered net, chiffon and lace cunningly employed. An English foulard on white ground, pin-spotted with black and having a design of pink roses, has a lovely collar of Irish lace and an inner waistcoat of silver embroidery. A white crêpe de chine is also lavishly trimmed with Irish lace, and the entire coat is made of the same beautiful material.

### Music at the Coronation.

The official programme of the music to be used at the Coronation service has now been issued, and is of an exceptionally interesting character. It embraces a period of five centuries of English Church music, from sixteenth-century Tallis to composers of the present day. The Litany, which is by Tallis, is the oldest composition selected, and will be sung immediately after the opening anthem. It was composed soon after the Second Prayer Book of Edward VI. in 1552. The threefold "Amen" was written by Orlando Gibbons, who was organist of Westminster Abbey some years after Shakespeare's time. Henry Purcell, who wrote two anthems for the coronation of James II., and was present at the enthronement of William and Mary, supplies the setting to Psalm III., and Handel's glorious anthem, "Zadok the Priest," will be used for the anointing. Three original and new works will be performed. Sir Hubert Parry has written "I was glad when they said unto me," and this will signalise the entrance of the King and Queen. Halfway through the anthem a pause will be made, so that the boys of Westminster School may exercise their ancient privilege of shouting from the gallery "Vivat Regina Alexandra" "Vivat Rex Edwardus." They will be answered by the choir below, who will take up the strain. The music for the actual crowning of the King will be by Sir W. Parratt, and Sir F. Bridge has composed the homage anthem "Kings shall see and arise."

### The New Ambassador to America.

The late Lord Pauncefote was one of the oldest members of the diplomatic service: he is succeeded by one of the youngest. The Hon. Michael Henry Herbert, C.B., who in future will act as His Majesty's Ambassador at Washington, is only 45. He is a brother of the Earl of Pembroke, the Lord Steward of the King's Household, and son of Lord Herbert Lea, who, as Mr. Sidney Herbert, was Secretary of State for War during the early part of the Crimean War. His statue stands in the courtyard of the department in Pall Mall. Mr. Herbert has held a number of important appointments, including those of Chargé d'Affaires and Secretary of Legation at Washington, and Secretary of Embassy at Paris. His wife is a New York lady, belonging to a well-known family in the States.

### Disastrous Fire in London.

A terrible loss of life through fire occurred on Monday evening about 5 o'clock in the heart of the City of London. No alarm came from the house itself, but a passer-by saw flames issuing from the windows of the third and fourth floors, occupied by the General Electric Company in Queen Victoria Street, and gave the alarm at the fire station. In a minute or so the engine and fire escape from Watling Street were in attendance, but unfortunately it was found that the escape did not nearly reach the girls, who stood at the windows shrieking for assistance. Before a taller ladder could be procured one man and several girls jumped a distance of 80 feet into the street below. A tarpaulin taken off a cart was held out by willing hands, and some of the girls were caught in this, but, overcome by fear, several jumped at once, and, naturally, with disastrous results. One of the firemen made two gallant rescues. Slung by a rope from a parapet he succeeded in passing through a window and bringing out two girls, one after another, who were safely conveyed to the street. But after the fire had been subdued the charred bodies of seven girls, aged between 15 and 17, and one boy were found, and several of those in hospital are seriously injured. The event seems the more sad because it appears that both of the two staircases in the house were available all the time.

### For Reading to the Sick.

THE POWER OF SYMPATHY.

THE look of sympathy, the gentle word,
Spoken so low that only angels heard;
The secret act of pure self-sacrifice,
Unseen by men, but marked by angels' eyes—
These are not lost.

The happy dreams that gladdened all our youth,
When dreams had less of self and more of truth;
The childhood's faith, so tranquil and so sweet,
Which sat like Mary at the Master's Feet—
These are not lost.

The kindly plan devised for others' good,
So seldom guessed, so little understood;
The quiet steadfast love that strove to win
Some wanderer from the ways of sin—
These are not lost.

Not lost, O Lord! for in Thy City bright,
Our eyes shall see the past by clearer light,
And things long hidden from our gaze below
Thou wilt reveal; and we shall surely know
These are not lost.

*Richard Metcalf.*

Love, true pure love towards God and towards man is the very essence and meaning of the Christian life. As the grace of God begins to change a man's heart he begins to grow in love, to lay aside self-seeking, and to live for others.

*F. Paget.*

Always say a kind word when you can, if only that it may come in, perhaps, with singular opportuneness, entering some mournful man's darkened room, like a beautiful firefly whose happy circumvolutions he cannot but watch, forgetting his many troubles.—*Arthur Helps.*

In helping others we benefit ourselves; we heal our own wounds in binding up those of others.—*S. Ambrose.*

Who shall attempt to describe the indescribable, and tell the power of sympathy? You go to see your friend on whom some great sorrow has fallen. You sit beside him, you look into his eyes. You say a few broken and faltering words, and then you go away disheartened. How entirely you have failed to do for him that which you went to do, that which you would have given the world to do; How you have seemed only to intrude on him, when you really longed to help him! How many times you have done this, and then how many times you have been afterwards surprised to find that you really did help him with that silent visit. Never let the seeming worthlessness of sympathy make you keep back that sympathy of which, when men are suffering around you, your heart is full. Go and give it without asking yourself whether it is worth while to give it. It is too sacred a thing for you to tell what it is worth. God, from whom it comes, sends it, through you, to His needy child.

*Phillips Brooks.*

It is a little thing to speak a phrase
Of common comfort, which by daily use
Has almost lost its sense; yet on the ear
Of him who thought to die unmourned, 'twill fall
Like choicest music.

*Talfourd.*

### Notes and Queries.

**The Editor is always willing to answer in this column, without any fee, all reasonable questions, as soon as possible.**
**But the following rules must be carefully observed:—**

1. **Every communication must be accompanied by the name and address of the writer.**
2. **The question must always bear upon nursing, directly or indirectly.**

**If an answer is required by letter a fee of half-a-crown must be enclosed with the note containing the inquiry, and we cannot undertake to forward letters addressed to correspondents making inquiries. It is therefore requested that our readers will not enclose either a stamp or a stamped envelope.**

*Home.*

(83) I should be glad if you could give me the address of a home in or about London where a lady suffering from slight chronic rheumatism could be received, either temporarily or permanently. She could afford to pay a sum not exceeding ten shillings weekly. —*K. B.*

The sum offered is very small, or the Ladies' Home, 53 Abbey Road, St. John's Wood, appears the most suitable for the case. The Woodside Home, Whetstone, N., is another home for which she might be eligible.

Can you tell me of a seaside nursing-home, preferably in Sussex, where we could send our maid for change of air and rest?—*A. Z.*

The Young Women's Christian Association's Home of Rest for Servants and Others, 32 Park Road, St. Leonard's-on-Sea, seems suitable.

My husband suffers from heart trouble, and is unable to follow his employment. Could you advise me how to obtain one or two children (over two if possible) to nurse? I cannot afford to advertise. Would you also tell me how much per month I ought to be paid?—*A. C.*

Your best plan seems to be to apply to the Secretary, Local Government Board, Whitehall, S.W., and ask for particulars of terms for receiving children boarded out by the Guardians. The payment is 4s. per week for food, 10s. per quarter for clothes, and 10s. a year for medical attendance. Information is supplied gratis.

Can you tell me of a home where a lady inebriate could be received, and where the fees are not very high.—*Anxiety.*

You will find a short list in "Burdett's Hospitals and Charities."

Can you tell me of a home where a lady between 50 and 60, well educated, but with no means, could be received? Her brother would, I think, be prepared to pay about £1 weekly for her. At times she is apparently quite well, and then she suddenly gets delusions.—*E. B.*

The National Association for Promoting the Welfare of the Feeble-Minded, 53 Victoria Street, S.W., would possibly be able to recommend a suitable private home.

*Eczema.*

(84) I suffer from eczema. Would that prevent my being a hospital nurse? It does not seem to affect my health.—*M. B.*

Even if your health continued good, it is most undesirable for the sick to be nursed by anyone suffering from a skin affection.

*Child's Nurse Training.*

(85) 1. Will you kindly give me the name of the institution at which children's nurses are trained? There is one in London I know, and another in Manchester. 2. At what time of the year should the names and information respecting trained nurses be sent for insertion in "Burdett's Official Nursing Directory"? What is the fee for insertion?—*Matron.*

1. The Norland Institute, 10 Pembridge Square, London, W., and the Liverpool Ladies' Sanitary Association, 8 Sandon Terrace, Liverpool, are the two best known institutions for this work. 2. Send the information at any time; it will be entered for the next issue of the book. There is no charge for insertion.

*Small-pox.*

(86) Can you tell me to whom to apply for an appointment as nurse on a small-pox ship? I am fully trained and also hold a certificate for fever nursing. 2. Can you give me the name of the training home for children's nurses?—*Manchester.*

Apply to the Clerk, Metropolitan Asylums Board, Victoria Embankment, W.C. 2. The Norland Institute, 10 Pembridge Square, W.

**Standard Nursing Manuals.**

"The Nursing Profession: How and Where to Train." 2s. net; post free 2s. 4d.
"Art of Massage." (Second Edition.) 6s.
"Elementary Physiology for Nurses." 2s.
"Elementary Anatomy and Surgery for Nurses." 2s. 6d.
"Practical Handbook of Midwifery." 6s.
"Surgical Ward Work and Nursing." Revised Edition. 3s. 6d. net; post free 3s. 10d.
"Mental Nursing." 1s.
"Art of Feeding the Invalid." 1s. 6d.
All these are published by the SCIENTIFIC PRESS, Ltd, and may be obtained through any bookseller or direct from the publisher, 28 and 29 Southampton Street, London, W.C.

### Travel Notes.

By Our Travelling Correspondent.

### CI.—WHAT WE CAN SEE FOR FIVE POUNDS.

### Bayeux.

Bayeux should be included in the week at Caen, from which place it is visited with ease. It is about seventeen miles distant, and either by cycle or train is nothing of a journey.

Bayeux is not so good a place to make your headquarters as Caen, most decidedly; but there is much to be seen which will occupy you an entire day. For myself the tapestry enthrals me to such an extent that, though I have visited the town very many times, I never fail to spend at least an hour studying that delightful chronicle of a bygone age.

There has been much controversy as to its origin. As most people know, it is a history, by means of a series of pictures executed in needlework, of Harold's visit to Duke William in Normandy, subsequent events in England, and finally the conquest of the country by the mighty Norman Duke. Popular belief ascribes the work to Queen Matilda and her ladies, and it seems very probable that such is the case.

In the long, dull days when reading, writing, and travelling were almost unknown, what leisure royal ladies must have had for endless embroidery! And the supposition that it could have been Saxon work seems absurd, for it would hardly be natural that the ladies of that conquered race should record their own humiliation..

Some authorities imagine it to be the outcome of an order of Odo, Bishop of Bayeux, half-brother of the Conqueror, whose companion he was on the expedition. Our historian Freeman shares this opinion, for he says, "I think no one can see the end of the battle—the House carls everyone lying dead in his harness, while the light-armed are taking to flight, some of them on the horses of the fallen—and not feel that he is in the presence of a work traced out by one who had himself seen the scenes which he thus handed down to later ages."

In one scene a dwarf is holding a horse, and it is thought he was the designer of the tapestry, and that he thus ingeniously represented himself among the stirring scenes.

### The Number of Figures.

In Bruce's guide to the tapestry, a most useful work, he enumerates the figures depicted, and thus they stand—623 men, 202 horses, 55 dogs, 505 other animals, 37 buildings, 41 ships, 49 trees—in all 1,512 figures.

What a stupendous labour it seems in these degenerate days, when our very pocket-handkerchiefs are hemmed by machinery!

M. Larousse gives a good, succinct description of this marvellous embroidery. In my translation I have been obliged to shorten his interesting account:—

"It represents the history of the conquest of England by William of Normandy in a series of scenes of which each subject is indicated by a Latin inscription. The series commences with the departure of Harold from the Court of Edward, and terminates at the battle of Hastings. The figures, drawn rudely and roughly, but full of force in their attitudes, are embroidered on a linen cloth with wools of eight different colours—light and dark blue, red, yellow, light and dark green, black, and fawn. These colours are far from being exact representations of the real subjects. In the figures the wool is laid flat and fastened with chain-stitch; the outlines, the joints, the folds of the clothing, are indicated by a kind of cord, and the lines of the flesh by a stroke of blue, red, yellow, or green.

"The historical scenes only occupy 33 centimetres, and are comprised between two borderings in which are real and fabulous animals, hunting scenes, episodes in rustic life, etc. . . . To facilitate, no doubt, the explanation of this long embroidered frieze, there is above and below more tapestry, equally ancient but less beautiful, 20 centimetres in depth, where are represented, instead of figures, simple crosses, in twos and threes, in front of a kind of altar; a ladder where the rungs terminate in a cross, and a little striped standard, the staff of which is surmounted by a cross."

In English figures the measurements are these: 200 feet long by 20 inches wide.

### The Explanatory Text.

This part always delights me; I wish I had room to quote the 58 inscriptions which fit (most correctly and effectively) the pictures. I like very much one where Harold is embarking for Normandy and wading through the sea to reach his boat, and carries a hound under his arm. Then where he is almost lost in a quicksand, at Mont St. Michel, is most realistic. By his great strength and agility he rescues two comrades. In 24, "Here Harold the Earl returned to England," there is a most quaint representation of people welcoming him from the windows. In 26 we have the interior of Westminster Abbey, with the hand of Providence indicating the spot where the Confessor should be buried. The events become a little mixed here, for in the next division the King (Edward) is still alive and addressing his faithful servants; but following that, lest there should be any mistake, we have him looking very shrunken and small, and the information "And here he is dead."

The royal lady (if, indeed, as I think there is strong reason to suppose was the case, Matilda was the artist) was naturally triumphant at the close, the last of the pictures being labelled "Et fuga verterunt Angli." And the English took to flight!

A small portion from the end has been lost, and it is supposed that it represented the Coronation of the Conqueror.

Throughout the English are represented with moustaches, and the Normans without; and there is always an attempt to give the same features and appearance in each representation of Harold and William.

When Napoleon was meditating an invasion of England he very naïvely caused this tapestry to be exhibited in different towns, with a view to inciting the populace to a more enthusiastic participation in his views of conquest.

### The Cathedral.

If you had not first seen the churches of Caen, the Cathedral of Bayeux would strike you as fine, and even in the neighbourhood of the Abbaye aux Hommes it is well worth seeing. I take particular interest in the crypt, because it holds in its depths all that is left of a long-past romance. Agatha, the Conqueror's youngest daughter, learnt to love the fair-haired Saxon Harold when he was on his embassy to her father's Court; but the course of true love did not run smooth, and she was betrothed against her will to Alfonzo of Spain. She prayed diligently, "until her knees were hardened," that she might die a maid, and her prayer was granted; she died but how or exactly where is not recorded on her journey into Spain to fulfil the marriage contract, and her dead body was brought back to Bayeux and placed in the crypt. There is very much more to tell you of this part of France, and I must devote another article to it in a few weeks. Arromanches is quite close; Falaise, too, and several more places of interest.

### TRAVEL NOTES AND QUERIES.

To Glasgow for a Week (M. G.).—You give no pseudonym, but I hope this will catch your eye. Yes; it can be done, even allowing the £1 16s. for the ticket. Write to Duncan's Temperance Hotel, Union Street, and ask for two rooms. Terms 7s. per day each, or 40s. for the week. You will find all the information you need in the *Nursing Section* for June 8th and June 15th in two articles I wrote on Glasgow and the neighbourhood.

South Coast of Devon or Cornwall (Olivia).—No; there is no charge for an answer in this column. Everything is dear by the sea in the season, but naturally cheaper the further you go from London, but then the journey costs heavily. Dartmouth is a favourite place with me because it is beautifully situated, and there are so many excursions to be made cheaply all round; third return ticket 34s., but there will be something cheaper in the tourist season. There are reasonable lodgings to be had. Go for one night either to the "Raleigh" or "Fairfax" Hotels, and look about for something suitable. Clovelly is delightful—nearest town, Bideford. There are many lodgings there, but full in the season; still, with management, reasonable accommodation can be found. In Cornwall, Fowey is charming, and still primitive; fare, third return, 42s. Tourist tickets much less in the season. This is a very nice place, and, I think, suitable for you. Most of the lodgings are on the Esplanade. Leave your luggage at the station, and go to seek lodgings. If you are too tired, there are several inns. Try "The Ship" for the night. There is a boarding house called "St. Catherine's," well spoken off, why not write and ask terms?

## Figures and Tables

**Fig. 45. f1:**
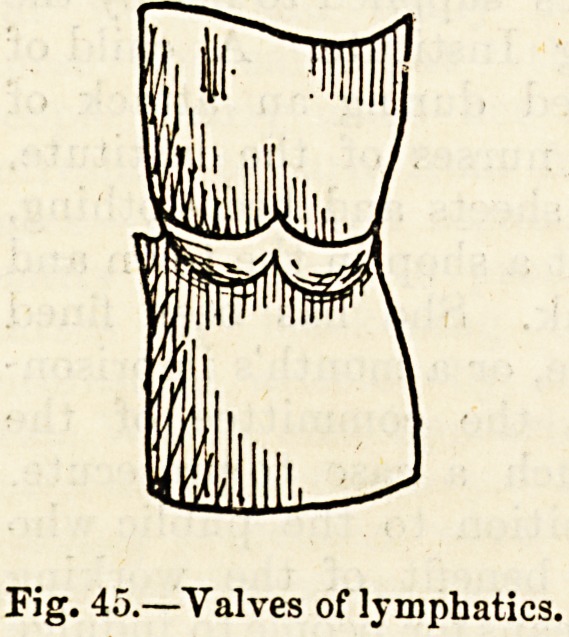


**Fig. 46. f2:**